# Next-Generation Sequencing-Based RiboMethSeq Protocol for Analysis of tRNA 2′-*O*-Methylation

**DOI:** 10.3390/biom7010013

**Published:** 2017-02-09

**Authors:** Virginie Marchand, Florian Pichot, Kathrin Thüring, Lilia Ayadi, Isabel Freund, Alexander Dalpke, Mark Helm, Yuri Motorin

**Affiliations:** 1 IMoPA UMR7365 CNRS-UL, Biopole Lorraine University, 54505 Vandoeuvre-les-Nancy, France; Virginie.Marchand@univ-lorraine.fr (V.M.); Florian.Pichot5@etu.univ-lorraine.fr (F.P.); Lilia.Ayadi@univ-lorraine.fr (L.A.); 2 Next-Generation Sequencing core facility, FR3209 BMCT CNRS-UL, Biopole Lorraine University, 54505 Vandoeuvre-les-Nancy, France; 3 Institute of Pharmacy and Biochemistry, Johannes Gutenberg University Mainz, 55128 Mainz, Germany; thuering@uni-mainz.de (K.T.); mhelm@uni-mainz.de (M.H.); 4 Department of Infectious Diseases, Medical Microbiology and Hygiene, Ruprecht-Karls University Heidelberg, 69120 Heidelberg, Germany; Isabel.Freund@med.uni-heidelberg.de (I.F.); Alexander.Dalpke@med.uni-heidelberg.de (A.D.)

**Keywords:** tRNA, 2′-*O*-methylation, RiboMethSeq, high-throughput sequencing, deleted strain, TrmH, TRM3

## Abstract

Analysis of RNA modifications by traditional physico-chemical approaches is labor intensive, requires substantial amounts of input material and only allows site-by-site measurements. The recent development of qualitative and quantitative approaches based on next-generation sequencing (NGS) opens new perspectives for the analysis of various cellular RNA species. The Illumina sequencing-based RiboMethSeq protocol was initially developed and successfully applied for mapping of ribosomal RNA (rRNA) 2′-*O*-methylations. This method also gives excellent results in the quantitative analysis of rRNA modifications in different species and under varying growth conditions. However, until now, RiboMethSeq was only employed for rRNA, and the whole sequencing and analysis pipeline was only adapted to this long and rather conserved RNA species. A deep understanding of RNA modification functions requires large and global analysis datasets for other important RNA species, namely for transfer RNAs (tRNAs), which are well known to contain a great variety of functionally-important modified residues. Here, we evaluated the application of the RiboMethSeq protocol for the analysis of tRNA 2′-*O*-methylation in *Escherichia coli* and in *Saccharomyces cerevisiae*. After a careful optimization of the bioinformatic pipeline, RiboMethSeq proved to be suitable for relative quantification of methylation rates for known modified positions in different tRNA species.

## 1. Introduction

RNA modification is a complex step in the post-transcriptional maturation of cellular RNAs and includes numerous chemical alterations of initially incorporated parental nucleotides. Among these various chemical reactions, the most frequent is the transfer of a methyl (CH_3_) group from a methylation donor (generally *S*-adenosyl-l-methionine, abbreviated as SAM or AdoMet) to different positions on nucleobases or to the 2′-OH of the ribose [1,2,3,4,5]. Methylated nucleotides are found in every studied RNA species, and their presence allows fine modulation (tuning) of RNA properties in different cellular processes [6,7,8,9]. One of the best-known RNA species is the transfer RNAs (tRNAs), whose properties and functions have now been studied for over 50 years. These relatively small RNA molecules have been extensively analyzed for their RNA modification profile, and a wealth of information is now available for tRNA from bacteria, lower eukaryotes (mostly yeasts and fungi) and mammals (human, rat, rabbit, and beef) [10]. This already served as a basis for extensive search of the corresponding enzymes and enzymatic machineries involved in the modification of tRNA species. Thanks to these efforts, most of the enzymes for tRNA modification in model organisms were identified and their specificity characterized [11,12,13]. It is noteworthy that archaeal species somehow escaped this thorough analysis, and only a few tRNA species are included in available databases. Archaeal enzymes are mostly characterized by sequence homology followed by functional tests to confirm the initial hypothesis on their predicted activity, as archaeal tRNA methyltransferase (aTrm56) [14,15].

One very common methylation in many RNA species is 2′-*O*-ribose methylation (2′-*O*-Me). This type of methylations may occur in all four canonical nucleotides in RNA and even many base-modified residues, the most frequent probably being 2′-*O*-methylated pseudouridine (ψm). 

### 1.1. Bacterial tRNA 2′-*O*-methylation

**Figure 1:**
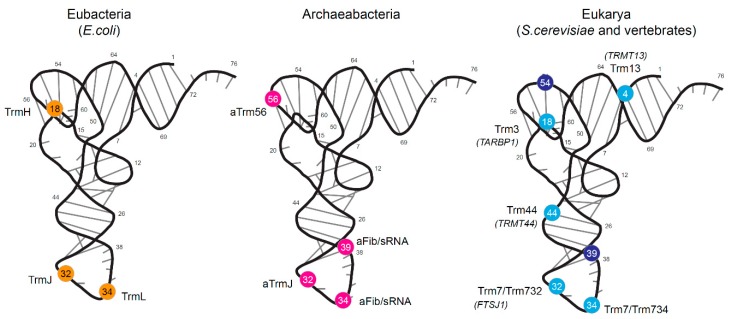
Known positions of tRNA 2′-*O*-methylations and corresponding enzymes in Eubacteria (*E. coli*/*Bacillus subtilis*), Archaea (data almost exclusively from *H. volcanii*) and Eukarya (*S. cerevisiae* and various vertebrates). tRNAs are represented in folded 3D structures. 2′-*O*-methylated nucleotides are shown as spheres, orange for Eubacteria, pink for Archaea. Light blue color corresponds to yeast modifications; additional vertebrate positions are shown in dark blue. Names of homologous human proteins are in italics.

Early on, the analysis of tRNA species from the model bacterial organism *Escherichia coli* demonstrated the presence of three possible positions for 2′-*O*-methylation (Table 1). A major site is located in the D-loop at position 18, where the highly conserved G residue is frequently converted to 2’-*O*-methylated guanine (Gm), while two others are located in the anticodon loop at positions 32 and 34 (wobble) where Cm and Um/cmnm^5^Um residues are frequently present (Figure 1). *E. coli* tRNA has no other reported sites, while other bacterial species, like *Bacillus subtilis* and *Mycoplasma capricolum*, seem to lack Gm18, but may contain Gm34 (*B. subtilis*) or cmnm^5^Um34 (*M. capricolum*) at the wobble position of the anticodon. 

The formation of Gm18 in bacterial tRNA is catalyzed by specific tRNA:Gm18-methyltransferases (MTases) from the SpoU (now renamed to TrmH) family, which had already been predicted to be an RNA:MTase [16,17,18], prior to experimental validation. This enzyme was found in the *gmk*-*rpoZ*-*spoT*-*spoU*-*recG* operon in *E. coli* [19] and was also conserved in related bacterial species and in the extreme thermophile *Thermus thermophilus* [20]. 

Recognition of tRNA substrates by TrmH was extensively investigated and the enzymatic mechanism elucidated by a structure-based site-directed mutagenesis study [21]. The TrmH protein from the thermophilic bacterium *Aquifex aeolicus* has been crystallized and its structure studied at high resolution [22]. 

Two other important sites of 2′-*O*-methylation in *E. coli* tRNA are located in the anticodon loop, at positions 32 and 34. The formation of Cm32 and Um32 tRNA^Ser^_1_ and tRNA^Gln^_2_ is catalyzed by YfhQ (renamed now to TrmJ or TrMet(Xm32)) [23], which belongs to the SPOUT family, in contrast to eukaryotic TRM7 from the RFM group (see below) [24].

Finally, the enzyme encoded by yibK, named TrmL, is a part of a more complex pathway [25] designed for the biosynthesis of a complex, multistep modification of the nucleotide at wobble position 34 in two leucyl isoacceptor tRNAs. Depending on the tRNA species, TrmL catalyzes the formation of Cm34 or Um34 in tRNA^Leu^(CmAA) and tRNA^Leu^(cmnm^5^UmAA) [26]. TrmL methylates pyrimidines, but not purine residues at the wobble position, and the 2′-*O*-methylation depends on prior N^6^-isopentenyladenosine modification at position 37.

In other bacteria, enzymatic tRNA modification systems targeting the ribose-2′-OH are only partially characterized. Recently, a tRNA MTase TrmJ in *Pseudomonas aeruginosa* PA14 was found to be involved in resistance to oxidative stress. TrmJ catalyzes the formation of Cm34, Um34 and, in contrast to *E. coli*, an additional Am34 in tRNA^Pro^(GGG) [27]. 

### 1.2. Archaeal tRNA 2′-*O*-Methylation

Archaeal tRNA 2′-*O*-methylation is only partially characterized up to now. In contrast to Bacteria, where only proteinaceous enzymes modify tRNAs, archaeal organisms use both stand-alone protein enzymes and C/D-box small nucleolar RNA (C/D snoRNA) guided machinery (Figure 1 and Table 1), sometimes with overlapping substrate and site specificity [28]. 

Methylation of the universally-conserved methylated position at position 32 in the tRNA anticodon in Archaea *Sulfolobus acidocaldarius* is catalyzed by a homolog of the bacterial TrmJ. However, while all four canonical nucleosides are substrates of the *E. coli* enzyme, the archaeal TrmJ can only methylate the ribose of a cytidine [24]. 

In contrast to Eubacteria, the methylation of the wobble residue C34 of *Haloferax volcanii* elongator tRNA^Met^ is guided by a box C/D RNA targeting the tRNA intron-containing precursor [29]. 2′-*O*-methylation of both nucleotides C34 and U39 in *H. volcanii* pre-tRNA^Trp^ also requires the box C/D ribonucleoprotein (RNP) machinery. Methylation occurs sequentially with the C/D-box small RNA (sRNA) guide in *trans*, which would also eliminate the obligatory refolding of the pre-tRNA before splicing [30]. Most euryarchaeal tRNA^Trp^ genes require a box C/D guide RNA within their intron for specifying methylation at these two sites [31]. 

The archaeal-specific modification Cm56 is formed by the SPOUT *S*-adenosylmethionine (AdoMet)-dependent MTase aTrm56, which is found only in Archaea [32,33]. aTrm56 is present in almost all of the archaeal genomes sequenced up to now, except in the crenarchaeon *Pyrobaculum aerophilum* in which the tRNA Cm56 2′-*O*-methylation is provided by a C/D sRNP [31]. 

### 1.3. Eukaryotic tRNA 2′-*O*-Methylation

In yeast and human, tRNAs 2′-*O*-methylations occurs at five rather conserved positions and two minor sites, the best conserved locations being Nm4, Gm18, Cm32/Nm34 and Um44 (Table 1).

The formation of the highly conserved Gm18 in *Saccharomyces cerevisiae* tRNAs is catalyzed by the enzyme encoded by ORF YDL112w (now renamed “Trm3”). Genetic depletion of YDL112w resulted in the complete absence of Gm18 in all tRNAs that naturally contain this modification. Other tRNA ribose methylations and the complex pattern of ribosomal RNA (rRNA) ribose methylations were not affected [34].

Anticodon 2′-*O*-modifications both at positions 32 (Cm32) and 34 (Nm34) in yeast tRNAs require a unique MTase Trm7 (encoded by YBR061C) [35], which also tightly interacts with ORF YMR259c (now named Trm732) for 2′-*O*-methylation of C32 and with Rtt10 (named Trm734) for 2′-*O*-methylation of N34 in substrate tRNAs [36]. Moreover, the presence of Trm7-catalyzed modifications in the tRNA anticodon loop is required for efficient modification of m^1^G at position 37, in the biosynthesis pathway leading to yW (wybutosine) [36,37]. Activities towards Cm32 and Nm34 seem to be independent, since a specific mutation in Trm7 selectively abolishes the formation of Gm34 only [38].

2′-*O*-methylated Um44 is frequent among eukaryotic cytoplasmic tRNAs bearing a long variable loop. Yeast ORF YPL030w (now renamed to Trm44) was identified by screening for the Um44:MTase activity of yeast genomic library of affinity-purified proteins. Consistent with the conservation of Um44 in eukaryotic tRNAs, Trm44 is found in metazoans and fungi, but surprisingly absent in plants [39].

The last 2′-*O*-methylation site in yeast tRNAs was found at position 4, and it is the only 2′-*O*-methylation that occurs in the middle of a duplex region in tRNA. It was shown that *S. cerevisiae* ORF YOL125w (renamed to Trm13) is required and sufficient for this modification [40]. 

The tRNA 2′-*O*-methylation profile in vertebrates is somehow similar to the one observed for lower eukaryotes, like yeasts, with conserved sites at position Cm/Um4, Gm18, Cm/Um/ψm32, Cm/Gm34 and Um44. One notable exception is the presence of Tm(m^5^Um)54 in the Tψ-loop; this nucleotide occasionally replaces the universally conserved T54 [41]. Another minor site is ψm39 (Figure 1 and Table 1).

In addition to common simple 2′-*O*-methylated residues, tRNAs from higher eukaryotes contain other more complex modified residues, where base modification is coupled with ribose methylation. For example, 2′-*O*-methyl-5-formyl modification of cytidine (f^5^Cm) was found at position 34 in bovine tRNA^Leu^ and confirmed for rabbit and lamb. Thus, f^5^Cm could be a general feature of cytoplasmic tRNAs^Leu^ in mammals [42]. 

Studies of higher eukaryotic RNA 2′-*O*-methylation enzymes have a long-standing history [43]. Already in the 1980s, the enzymatic activity acting at Gm34 in tRNA^Phe^ was detected using microinjection into *Xenopus laevis* oocytes [44]. However, systematic identification of the corresponding enzymes has started only recently. 

The only higher eukaryotic tRNA-specific 2′-*O*-MTase characterized up to now is a human homolog of yeast Trm7. Mutations in human FTSJ1, the Trm7 homolog, are present in two genetically-independent cell lines of patients and cause non-syndromic X-linked intellectual disability (NSXLID). Cells with loss-of-function FTSJ1 mutations nearly completely lack Cm32, as well as Gm34; moreover, an NSXLID patient with a novel FTSJ1-p.A26P missense allele specifically lacked Gm34, while levels of Cm32 and o^2^yW37 remained normal [38].

A homology search using yeast tRNA:MTases as probes revealed the presence of predicted homologs for all four known yeast tRNA:2′-*O*-MTases [5] (see Table 1); however, the activity of most of these proteins was not experimentally confirmed up to now. 

### 1.4. Functions of tRNA 2′-O-Methylation in tRNA Stability and Immunostimulation

Ribose 2′-*O*-methylation has been known for a long time to affect the stability and physico-chemical properties of the RNA chain. 2′-*O*-methylation of pyrimidines stabilizes the C3′-*endo* sugar conformation (and thus, the A-type RNA helix) primarily due to the steric repulsion among the 2-carbonyl group, the 2′-*O*-methyl group and the 3′-phosphate group in the C2′-*endo* form [45].

Archaea-specific hypermodified residues N^4^-acetyl-2′-*O*-methylcytidine (ac^4^Cm), 5-methyl-2′-*O*-methylcytidine (m^5^Cm) and N^2^-dimethyl-2′-*O*-methylguanosine (m^2^_2_Gm) were found to stabilize the C3′-*endo* form and therefore cause “conformational rigidity” as attested by proton Nuclear Magnetic Resonance spectroscopy. In particular, the ac^4^Cm was found to be extremely rigid due to additive effects of the N^4^-acetylation and 2′-*O*-methylation [46].

In addition to purely structural roles and general protection of RNA molecules from alkaline hydrolysis, tRNA 2′-*O*-methylation has an important functional role in fine-tuning of bacterial co-habitations with human (and other mammalian) organisms. It was noticed a long time ago that preparations of *Salmonella typhimurium* tRNA and rRNA provide RNA-dependent immunogenic activity [47]. More recently, it was clearly demonstrated that immunostimulation by tRNA is inhibited by tRNA:Gm18-MTase activity and even by the single methyl group on the 2′-oxygen of Gm18 as a natural modification in native tRNA [48,49]. This might explain differential immunostimulatory effects of different bacterial strains, and it was discussed that tRNA Gm18 modification might be used as an immune evasion mechanism. Of note, Gm18-modified tRNA acted as a TLR7 antagonist and blocked activation by RNA from plasmacytoid dendritic cells [47,48]. 

In addition, Gm18 also potently inhibited TLR7-independent human monocyte activation by RNA derived from a variety of bacterial strains. Antagonizing effects of Gm18-modified RNA are due to competition with stimulatory RNA for receptor binding. The effect of immunostimulation/suppression mediated by Gm18 in *E. coli* tRNA was observed for human and murine innate immune receptors TLR7/8, whereas the mouse-specific receptor TLR13 was insensitive to tRNA Gm18 [50], but inhibited by N^6^-methylation of rRNA (A2085 in *Staphylococcus aureus*/A2058 *E. coli*) [51].

### 1.5. Analytical Methods for Detection of 2′-O-Methylation in RNA

Despite the growing interest in the detection and quantification of 2′-*O*-methylated residues, no rapid and high-throughput analytical technologies have been applied to tRNAs, mostly due to their small size, very stable secondary and tertiary structure and an important proportion of other modified RNA residues. Reverse-transcription (RT) at low desoxynucleotide triphosphates (dNTP) concentrations is not very suitable for tRNAs, since natural RT-pauses are frequent, and RT-arresting modified residues prevent the generation of long primer extension products [52,53]. High-pressure liquid chromatography (HPLC) and HPLC coupled with mass spectrometry (MS)-related techniques require purification of tRNA species to near homogeneity, thus limiting their application to rather global analysis of the RNA modification profile [54,55,56]. Other approaches were also proposed, but did not become popular in the field [57]. 

Recently, high-throughput techniques have been developed for the analysis of a small subset of RNA modifications, namely m^6^A, m^1^A, pseudouridine and m^5^C [58,59,60,61,62]. These techniques allow transcriptome-wide screening, but the quantification of modification is uncertain and, when possible, requires complex calibration procedures. Only one type of modification in RNA can be detected and precisely quantified by next-generation sequencing (NGS), due to the induced resistance of the phosphodiester bond to cleavage in the presence of a neighboring 2′-*O*-Me group [63,64,65]. Thus, the measurement of protection against nucleolytic cleavage provides an excellent and quantitative way to measure 2′-*O*-modification.

The aim of the present study was to adapt the previously-published RiboMethSeq protocol [65] to 2′-*O*-methylation analysis of tRNAs. The main difficulties are related to the short length of most tRNA species, their stable 2D and 3D RNA structures, the existence of very similar, closely-related tRNA isoacceptors and the presence of numerous other modified nucleotides affecting both the efficiency of RNA cleavage, as well as primer extension by RT. 

## 2. Results and Discussion

We first evaluated the results from the application of the standard RiboMethSeq protocol (previously adapted to rRNA) for comparatively short tRNA sequences from *E. coli* and *S. cerevisiae*. The RiboMethSeq protocol [65] is based on high-throughput analysis of an alkaline RNA digest and is able to precisely map resistant phosphodiester bonds, with such sites mostly corresponding to natural 2′-*O*-methylated 5′-adjacent riboses. However, spontaneous structure- or sequence-dependent resistance to alkaline hydrolysis may generate false-positive hits in the analysis. 

**Figure 2:**
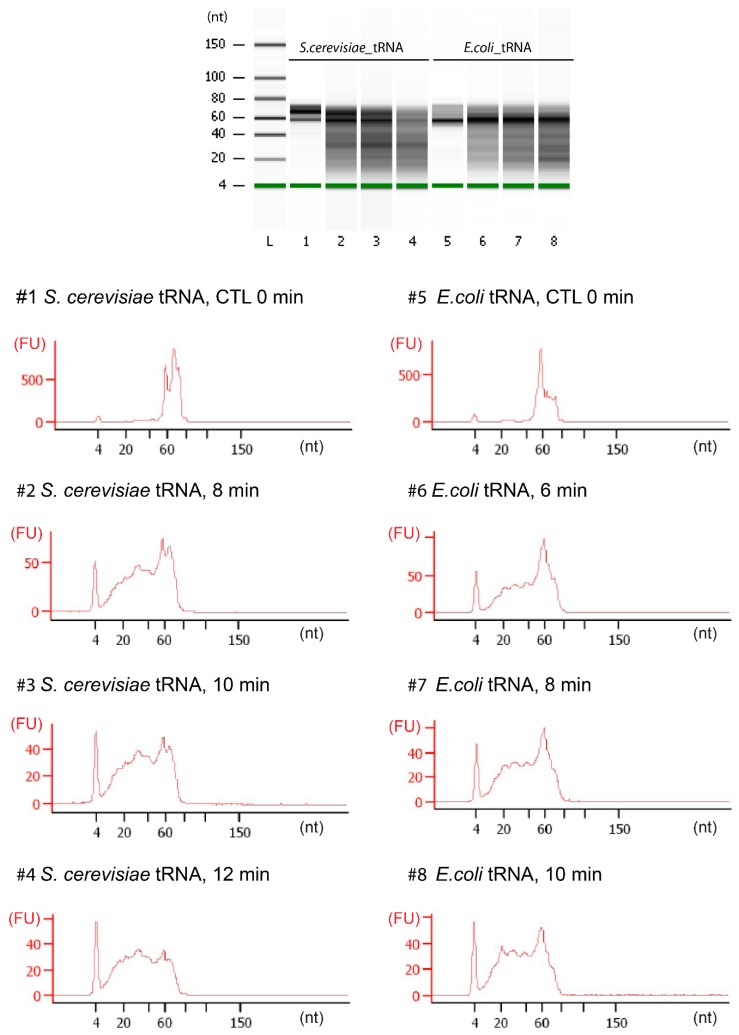
Fragmentation profiles for *E. coli* and *S. cerevisiae* total tRNA fractions. Alkaline hydrolysis was performed for 6–12 min (as indicated on the traces). Fragments size was analyzed by capillary electrophoresis on a Bioanalyzer 2100 (Agilent, Santa Clara, CA, USA) Small RNA Chip. FU: fluorescence unit.

One essential step of the RiboMethSeq protocol consists of the generation of a suitable library of RNA fragments after alkaline treatment. Short and structured RNA species (like tRNAs) are comparatively resistant to alkaline hydrolysis, and their fragmentation conditions have to be optimized to get sufficient amounts of short 20–40-nt fragments. Preliminary tests indicated that hydrolysis times of 6–10 min were appropriate for *E. coli* tRNAs, while a somewhat longer treatment of 8–12 min was required for the *S. cerevisiae* tRNA fraction (Figure 2). These tRNA fragments were converted to libraries and subjected to single-read Illumina sequencing for 50 nt.

With standard treatment parameters, the RiboMethSeq procedure uses reads of a minimum of 17 nt in length, which are aligned to the reference sequence with the end-to-end option. Only 5′-end extremities are used for analysis with calculation interval of six neighboring nucleotides. The application of these parameters for tRNA 2′-methylation analysis demonstrated, in principle, the detection of modified nucleotides, but also showed the necessity for a careful optimization of all treatment steps of the analysis pipeline. 

The necessity for optimization is related to short and quite similar tRNA genes (transfer DNA, tDNA) sequences used for alignment, to the very irregular alkaline cleavage profile of these small and strongly-structured RNA molecules and certainly to the presence of numerous other modified nucleotides with RT-arresting properties, decreasing even further the number of available sequencing reads at certain tDNA regions.

**Figure 3:**
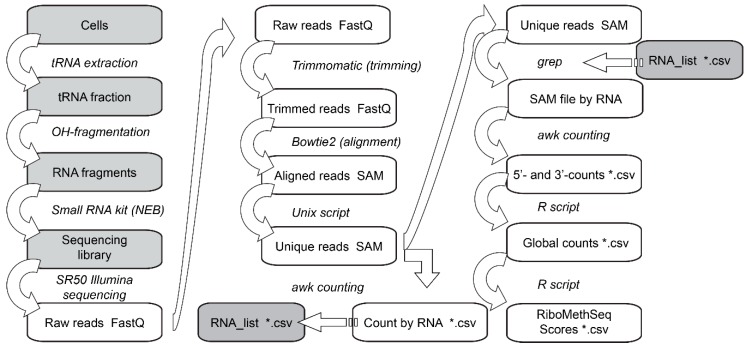
Optimized treatment pipeline for tRNA 2′-*O*-methylation analysis by high-throughput sequencing. SAM: sequence alignment/map.

Taking into account all of these difficulties, we proceeded with the optimization of all treatment steps (Figure 3), the most important concerning the used read length, selection of only uniquely aligned reads, simultaneous usage of 5′- and 3′-end information, as well as the calculation of scores for shorter neighboring regions and, finally, a careful optimization of the reference tDNA dataset. All of these optimization steps are discussed below.

### 2.1. Construction of the Reference tDNA Dataset

**Figure 4:**
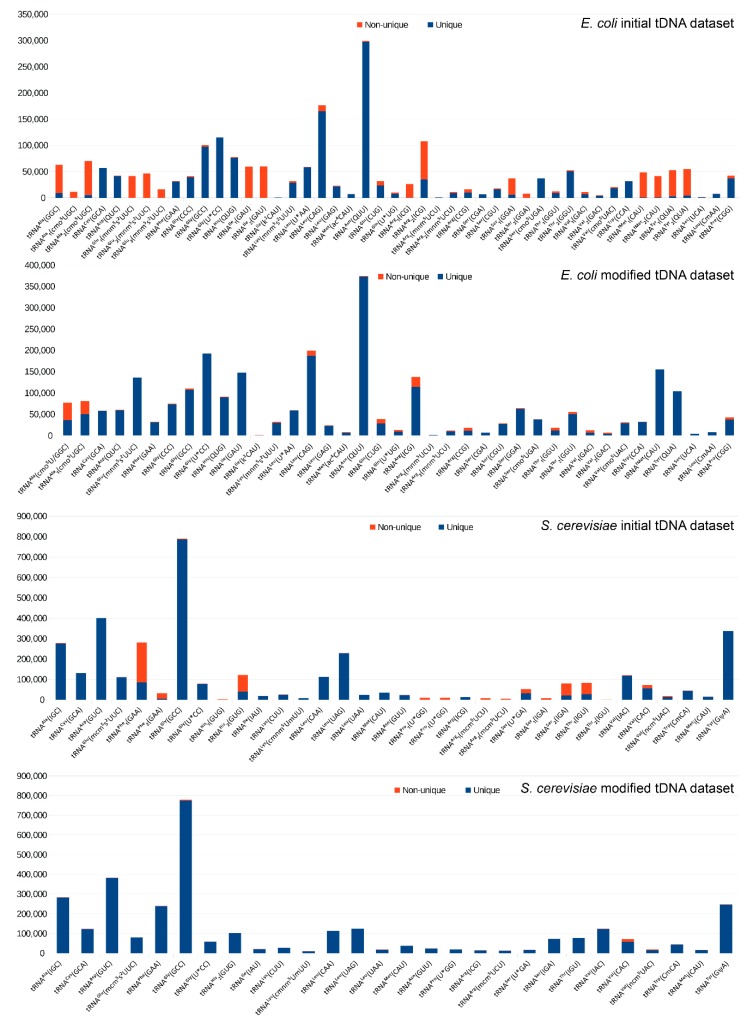
Proportion of non-uniquely mapped reads with initial and collapsed (optimized) transfer DNA (tDNA) datasets for each tRNA species. For both datasets, the number of uniquely and non-uniquely mapped reads was calculated for each tRNA species and plotted as stacked bars.

Initial tDNA datasets for alignment were constructed from sequences of modified tRNA species deposited in tRNA database (tRNAdb) [66], converted to DNA sequences in Fasta format. Inspection of the sequencing reads alignment to this tDNA reference revealed that, even at a minimum length of 17 nt, a significant number of reads mapped to multiple locations in tRNAs (Figure 4). This was mostly due to almost identical tDNA species present in the dataset and also to the presence of highly repetitive regions. In order to obtain better certitude in read mapping, we first decided to reduce and simplify the tDNA reference datasets for analysis. 

In order to identify closely-related tDNA sequences, the Levenshtein distance was calculated for the complete tDNA dataset for known modified tRNA species, obtained from tRNAdb. 

Distances between tDNA sequences are given in Figure S1; the calculation was performed for the dataset before and after collapsing of closely-related sequences. tDNA sequences with Levenshtein distance <4 (corresponds to a maximum of three nucleotide replacements) were collapsed into one unique sequence, and an undefined nucleotide “N” was placed at the divergent positions. Other closely-related pairs were retained in the dataset. At the same time, observed sequencing non-conformities and errors were corrected, and instances of the A-derived, but C-base pairing modified nucleotide inosine (I) present in some tRNA species were replaced by a G in the reference tDNA dataset. The same strategy was applied to *E. coli* and *S. cerevisiae* tDNA datasets. Details of the adaptation of both tRNA datasets are given in Tables S1 and S2.

### 2.2. Alignment and Analysis

For alignment to optimize the tDNA dataset, raw read trimming was performed to keep all sequences >8 nt. Of note, this length, while unsuitable for large genomic alignments, is still appropriate for a limited size tDNA “genome”. In order to gain additional information on both the 5′- and the 3′-end positions for every aligned read, only trimmed (with adapter removed) sequences were retained for subsequent alignment. This reduced the number of usable reads, but still provided some gain in information, since both 5′- and 3′-end locations were used.

Bowtie2 alignment parameters were adjusted for short input sequences (L = 10) in end-to-end mode. In order to avoid contamination by ambiguously (multiply) mapped sequencing reads, only uniquely-aligned sequences were taken for further analysis. Details for the number of retained reads at every step are given in Table S3. The positions of the 5′- and 3′-ends of every read were extracted after conversion of the aligned *.sam file to bed format with bedtools. 

The positions of the 5′- and 3′-ends were cumulated together to obtain the global end-coverage profile, and these data were used to calculate RiboMethSeq scores (ScoreMean (derived from ScoreMax) and ScoresA, B and MethScore(C)) as described previously [63,65]. Since the length of tRNA sequences is rather limited and the cleavage profile of tRNAs is highly irregular, only two neighboring nucleotides on each side were taken into account (instead of six in the standard RiboMethSeq approach).

As a result of this optimization, over 95% of trimmed reads were unambiguously mapped to tDNA reference sequences, and a better homogeneity of the coverage was obtained. All tRNAs (except one species in *E. coli* and one in *S. cerevisiae*) received sufficient coverage, and further analysis of 2′-*O*-methylation could be performed.

### 2.3. Profiles for tRNAs from WT and ΔTrmH *E. coli* Mutant Strains

Analysis of *E. coli* tRNA 2′-*O*-methylation was performed in parallel for the wild-type (WT) strain and for a strain with the deletion of the nonessential *trmH* gene responsible for Gm18 formation in bacterial tRNAs. *E. coli* tRNAs were reported to contain 2′-*O*-methylated residues at three different locations (Gm18, Cm32 and Cm/Um/cmnm^5^Um34; see the Introduction). As expected, RiboMethSeq efficiently detected alkali-resistant positions, which mostly correspond to 2′-*O*-methylations, to other modified RNA residues affecting cleavage or phosphorylation/ligation efficiency for the adapter or to naturally insensitive non-modified residues. The present goal was to provide a set of potentially modified positions to be verified by independent approaches and also to gain relative quantification of the methylation level for known 2′-*O*-methylated nucleotides.

**Figure 5:**
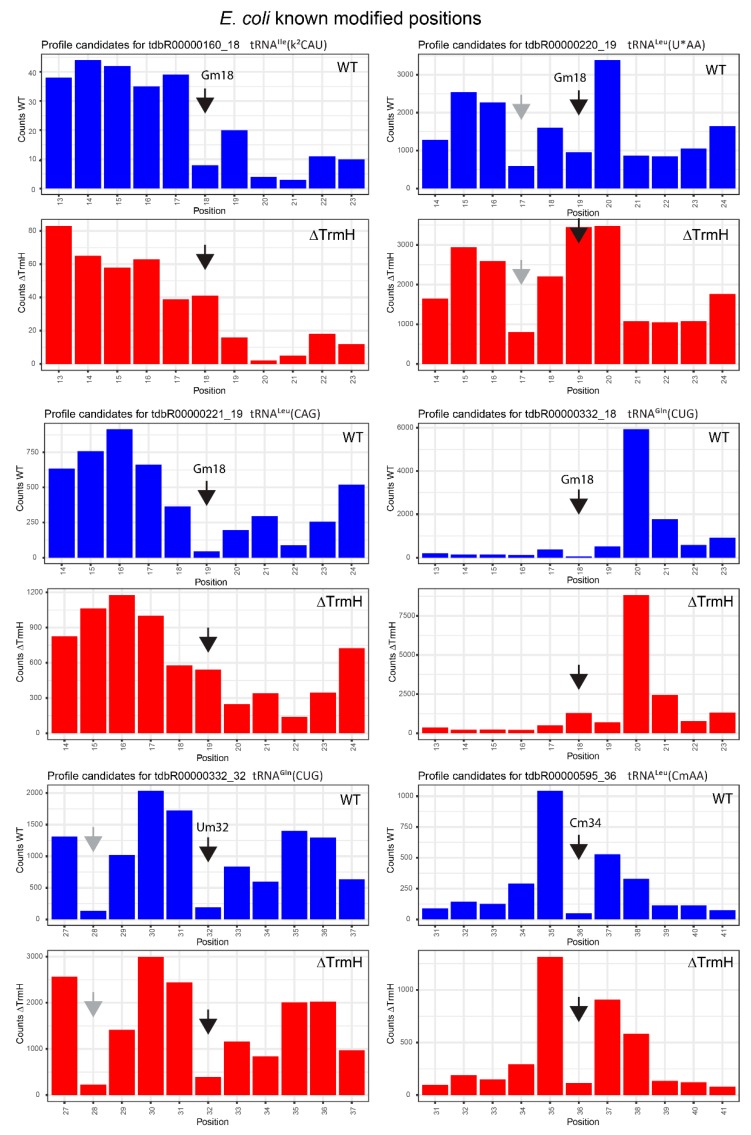
Representative cleavage profiles for six *E. coli* tRNA species for wild-type (WT) and ΔTrmH strains. The cumulated number of the 5′- and 3′-end extremities at every position is traced as vertical bars. All profiles are centered for the known 2′-*O*-methylated position shown by the black arrow; false-positive alkali-resistant positions are indicated by gray arrows.

Representative cleavage profiles for the selection of well-detectable 2′-*O*-methylated sites are shown in Figure 5, and the corresponding scores are listed in Table 2. All previously reported 2′-*O*-methylated sites gave rather high values of ScoreMean, while ScoreA [63] varied considerably. The comparison of both scores obtained for WT and ΔTrmH strains demonstrated that the value of the score considerably decreased for all TrmH-dependent 2′-*O*-methylations (Gm18), but remained very stable for all 2′-*O*-methylated residues at positions 32 and 34, catalyzed by TrmJ and TrmL (Figure 1 and Table 1). Inspection of the corresponding cleavage profiles confirmed that cleavage efficiency at all TrmH-catalyzed positions drastically increased in the ΔTrmH strain. This clearly demonstrated that tRNA 2′-*O*-methylation can be detected and even precisely quantified using RiboMethSeq, even when the RNA cleavage profile is highly irregular. However, multiple false-positive hits were also detected in various tRNAs (Figure S2), some of them in proximity to highly accessible tRNA nucleotides or to some of the numerous modified residues in tRNA, namely pseudouridine or m^7^G46. 

*E.* coli tRNA^Met^(ac^4^CAU), tdbR00000276 (highlighted in grey see Table 2), was previously reported to contain at least partial Gm18 methylation. Analysis of the RiboMethSeq results for this tRNA demonstrated that rather low scores were obtained for potential Gm18, and in addition, no difference was detected between WT and ΔTrmH strains. In order to check the real existence of Gm modification in this tRNA, it was purified to near homogeneity by hybridization with biotinylated DNA oligonucleotide and subjected to nucleotide analysis by HPLC-MS/MS (Figure S3a,b). As clearly seen in the figure, only trace amounts of Gm were detected, confirming the absence of Gm18 in *E. coli* tRNA^Met^(ac^4^CAU). Following up on our previous studies on the immunostimulatory effect of a Gm18 modification in *E. coli* tRNA [48], we tested the native tRNA for its effect on TLR7-mediated stimulation of interferon emission from peripheral blood mononuclear cells (PBMCs). Indeed, the native tRNA induced the substantial release of α-interferon, which would be in contrast to the possible presence of Gm18, but is in excellent agreement with its absence (Figure S3c). These findings illustrate that the mapping of ribose methylated residues in tRNA is accurate to a point that reports from the literature can be efficiently corrected. 

### 2.4. Analysis of 2′-*O*-Methylation in WT and ΔTRM3 Yeast Mutant Strains

**Figure 6:**
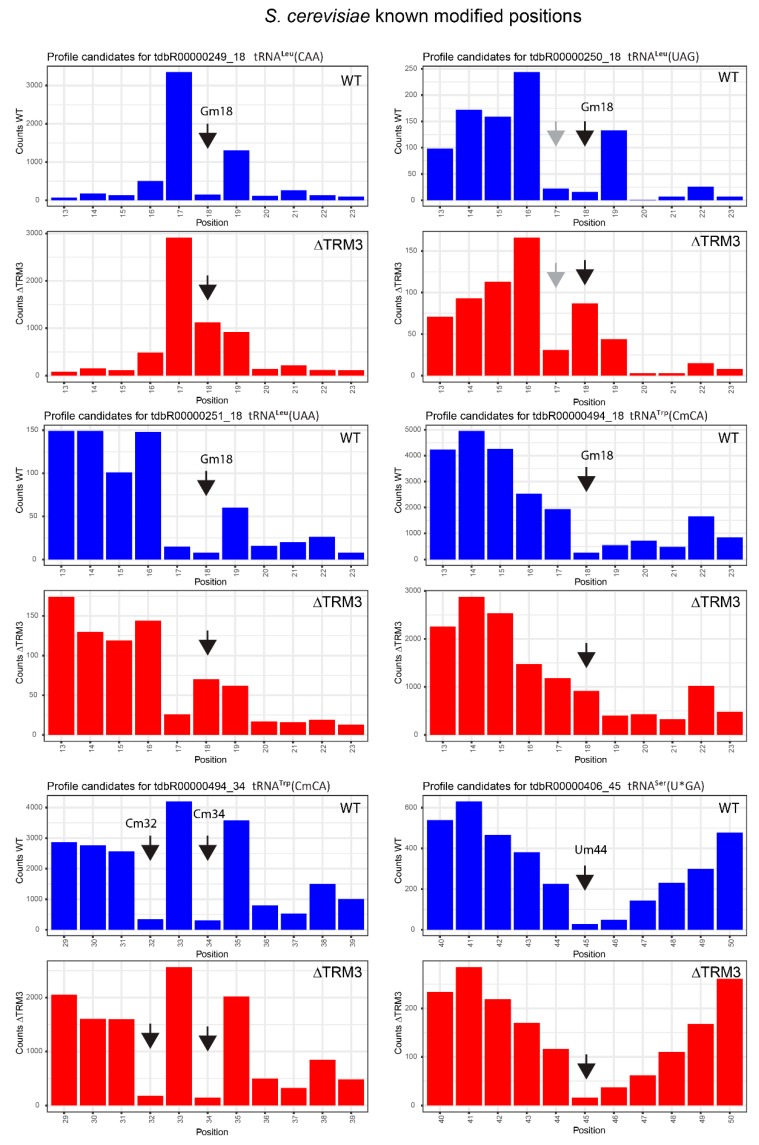
Representative cleavage profiles for six *S. cerevisiae* tRNA species for WT and ΔTRM3 strains. The cumulated number of the 5′- and 3′-end extremities at every position is traced as vertical bars. All profiles are centered for the known 2′-*O*-methylated position shown by the black arrow; false-positive alkali-resistant positions are indicated by gray arrows.

The optimized treatment pipeline described above was also applied for the analysis of alkaline hydrolysis-resistant positions in yeast total tRNAs extracted from WT and ΔTrm3-strains. All 19 known positions of 2′-*O*-Me in yeast gave strong and consistent values of ScoreMean (median value 0.93, standard deviation (SD) 0.077), as well as MethScore (median 0.83, SD 0.15). All scores for Gm18 demonstrated a considerable decrease (average ScoreMean −0.037 and MethScore −0.1) in the ΔTrm3 yeast strain compared to the WT cells. In contrast, the calculated values of the scores for other known 2′-*O*-methylated positions (Nm5-6, Nm32–34, Nm45) were not at all affected in the ΔTrm3 strain (see Table 2). Representative cleavage profiles are given in Figure 6 and false-positive hits in Figure S4.

### 2.5. Limitations

The results obtained in this study demonstrate that RiboMethSeq can be successfully applied not only to rRNA sequences in different species, but also to tRNAs, including also relative quantification of 2′-*O*-methylation levels in this type of RNA. However, rather important sequencing coverage is required for reliable analysis, and the cleavage profiles for tRNAs remain extremely irregular and, thus, generate an important number of false-positive hits. Some of them correspond to other modified tRNA nucleotides, and independent approaches are required to distinguish real RNA modification and phosphodiester bonds that are alkaline resistant for other reasons, such as three-dimensional structure. The ScoreMean parameter provides rather good sensitivity (all known 2′-*O*-methylated positions have a high ScoreMean value), but poor selectivity, while the opposite is true for ScoreA. Other proposed scoring schemes are even less discriminative and were not used here.

The use of cell lines or strains devoid of selected RNA-modification enzymes provided considerable support in the distinction between truly ribose-methylated positions from false-positive hits, since only signals corresponding to real modified nucleotides are affected, while signals for false-positives and other unaffected modified nucleotides remain generally stable. For a more precise quantification of the RNA modification level, the calibration of RiboMethSeq signals with unmodified RNA species extracted from the deleted or knockout strain is strongly recommended.

## 3. Materials and Methods 

### 3.1. tRNA Sources

The *S. cerevisiae* parental strain (Y00000, strain BY4741; S288C isogenic yeast strain: MATa; his3Δ1; leu2Δ0; met15Δ0; ura3Δ0) and MTase mutant (Y03809, MATa; ura3Δ0; leu2Δ0; his3Δ1; met15Δ0; YDL112w::kanMX4) deficient for Trm3 (Gm18-2′-*O*-MTase) were obtained from Euroscarf, Johann Wolfgang Goethe-University Frankfurt and grown at 30 °C in yeast extract-peptone-dextrose (YPD) medium (Carl Roth, Karlsruhe, Germany) to mid-log phase.

The *E. coli* parental strain (BW25113; CGSC#7636; F-, Δ(araD-araB)567, ΔlacZ4787(::rrnB-3), λ, rph-1, Δ(rhaD-rhaB)568, hsdR514) and trmH (Gm18-2′-*O*-MTase) deficient mutant (JW3626-1; CGSC#11693; F-, Δ(araD-araB)567, ΔlacZ4787(::rrnB-3), λ-, rph-1, ΔtrmH755::kan, Δ(rhaD-rhaB)568, hsdR514) were purchased from *E. coli* Genetic Stock Center CGSC, Yale University, and grown to mid-log phase at 37 °C in Luria-Bertani (LB) medium (AppliChem, Darmstadt, Germany). Knockout strains were grown with 200 μg/mL geneticin (G418) (Carl Roth) and 50 μg/mL kanamycin (AppliChem), respectively.

### 3.2. Isolation of tRNA 

For optimal amounts of RNA, bacteria and yeast were pelleted and frozen at −80 °C overnight and thereafter lysed in 40 mg/mL lysozyme (Serva, Heidelberg, Germany) or 50 U/mL lyticase (Sigma Aldrich, St. Louis, MO, USA), respectively. Total yeast and bacterial RNA were isolated using TRIzol reagent (Thermo Fisher Scientific, Waltham, MA, USA) according to the manufacturer’s instructions. Total RNA preparations were separated by denaturating PAGE (10% acrylamide, 8 M urea); tRNA-containing bands were cut out and shaken in 0.5 M sodium acetate overnight. tRNA was precipitated with ice cold ethanol and resuspended in nuclease-free water. The concentration of RNA was controlled via UV absorbance.

### 3.3. tRNA Fragmentation and Analysis

tRNA (about 100 ng) hydrolysis was performed in 50 mM bicarbonate buffer pH 9.2 for different times (6–10 min for *E. coli* or 8–12 min for *S. cerevisiae*) at 95 °C. The reaction was stopped by ethanol precipitation using 3 M Na-acetate, pH 5.2 and glycoblue as a carrier in liquid nitrogen. After centrifugation, the pellet was washed with 80% ethanol and resuspended in nuclease-free water. The sizes of generated RNA fragments were assessed by capillary electrophoresis using a Small RNA chip on the Bioanalyzer 2100 (Agilent).

### 3.4. Library Preparation

RNA fragments without any gel-purification step were directly 3′-end dephosphorylated using 5 U of Antarctic phosphatase (New England Biolabs, Ipswich, MA, USA) for 30 min at 37 °C. After the inactivation of the phosphatase for 5 min at 70 °C, RNA fragments were phosphorylated at the 5′-end using T4 polynucleotide kinase (PNK) and 1 mM ATP for 1 h at 37 °C. End-repaired RNA fragments were then purified using the RNeasy MinElute Cleanup kit (QIAGEN, Hilden, Germany) according to the manufacturer’s recommendations, except that the volume of 96% ethanol was adjusted for RNA binding. Elution was performed in 10 µL of nuclease-free water. RNA fragments were converted to the library using the NEBNext Small RNA Library kit (ref#E7330S, New England Biolabs; or equivalent from Illumina, San Diego, CA, USA) following the manufacturer’s instructions. DNA library quality was assessed using a High Sensitivity DNA chip on a Bioanalyzer 2100. Library quantification was done using a fluorometer (Qubit 2.0 fluorometer, Invitrogen, Waltham, MA, USA).

The library mix for sequencing was adjusted to obtain ~10–15 millions of raw reads for each library; this number was found to be sufficient for a 5000–8000 nt length reference sequence (end-coverage ~1000×). 

### 3.5. Sequencing

Library sequencing was performed using the Illumina HiSeq 1000 sequencer in a single read mode for 50 nt with SBS kit v3. Primary analysis of sequencing quality was done with Illumina RTA 2.12 software, to insure a >Q30 quality score for >95% of the obtained sequences. Following the SR50 sequencing run, demultiplexing was done with Illumina BclToFastq v2.4; reads not passing the quality filter were removed.

### 3.6. Data Analysis

Raw reads after demultiplexing were trimmed to remove the sequence of the Illumina 3′ adapter. Trimming was performed with Trimmomatic v0.32 [67] with the following parameters: -phred33 /adapters/TruSeq3-SE.fa:2:30:10 LEADING:30 TRAILING:30 SLIDINGWINDOW:4:15 MINLEN:8 AVGQUAL:30. Tests with a training dataset demonstrated that with stringency parameter = 10, Trimmomatic removes the adapter with a minimal size of 16 nts. Thus, only trimmed and adapter-free reads of <35 nt were taken for alignment. Alignment to the reference tDNA sequences was performed with Bowtie2 ver2.2.4 [68] in end-to-end mode using the following parameter set: --no-1mm-upfront -D 15 -R 2 -N 0 -L 10 -i S,1,1.15. Preliminary tests demonstrated that the use of soft trimming during alignment (-- local settings) is not recommended, since the 5′- and 3-end may be incorrectly determined. Selection of only uniquely-mapped reads (single reported alignment position in the reference sequence) in the resulting *.sam file was done using values of the NM and XS fields (grep -E “@|NM:” *.sam|grep -v “XS:”). Uniquely-mapped reads were extracted from the *.sam file by RNA ID and converted to *.bed format using bedtools v2.25.0 [69]. Positional counting of the 5′-and 3′-ends of each read was performed with the awk Unix command. Further treatment steps were done in the open-source R environment (v3.0.1) [70]. In brief, 5′-end and 3′-end counts were merged together by RNA position and used for the calculation of ScoreMean (derived from the ScoreMax described previously), as well as Scores A and B [63] and MethScore [65]. Scores were calculated for 2 neighboring nucleotides instead of 6 in the standard RiboMethSeq procedure. Profiles of RNA cleavage at selected (candidate and previously known) positions were extracted and visually inspected. 

## 4. Conclusions

The alkaline hydrolysis-based RiboMethSeq method (Figure 3) can be successfully applied to the analysis of known tRNA 2′-*O*-methylations and their dynamics or variations under different conditions. Quantification of the modification level is possible with careful calibration, obtained either with unmodified tRNAs extracted from a relevant KO strain or, if not available, with a synthetic RNA transcript with an identical sequence. The candidates for novel modified positions revealed by this high-throughput technique should be further validated by orthogonal approaches, either by physico-chemical methods, like HPLC-MS/MS analysis or by primer extension at low dNTP concentrations, an approach specific for RNA 2′-*O*-methylations. In the case of Gm18 in *E. coli* tRNA^Met^(ac^4^CAU), where we have exercised such a validation, the RiboMethSeq method proved accurate and led to the correction of the data from the literature. As this example has demonstrated, RiboMethSeq may be used for the correct prediction of the immunosilencing properties of RNA molecules based on the functional effect of ribose methylation on the recognition by components of the innate immune system. 

## Figures and Tables

**Table 1:** Known transfer RNA (tRNA):2′-*O*-methyltransferases in model organisms (*Escherichia coli*, *Haloferax volcanii*, *Saccharomyces cerevisiae*, *Homo sapiens*) and their tRNA substrates.

Organism	Enzyme	Enzyme Alt. Name	Position	Modification	tRNA	Anticodon	tRNAdb Reference
*E. coli*	TrmH	spoU	18	Gm	Ile	k^2^CAU	tdbR00000160
			18		Leu	cmnm^5^UmAA	tdbR00000220
			18		Leu	CAG	tdbR00000221
			18		Leu	GAG	tdbR00000222
			18		Met	ac^4^CAU	tdbR00000276
			18		Gln	CUG	tdbR00000332
			18		Gln	U*UG	tdbR00000333
			18		Ser	CGA	tdbR00000390
			18		Ser	GGA	tdbR00000392-3
			18		Ser	cmo^5^UGA	tdbR00000394
			18		Tyr	QUA	tdbR00000545-46
			18		Leu	CmAA	tdbR00000595
	TrmJ	YfhQ, TrMet(Xm32)	32	Cm	Ser	cmo^5^UGA	tdbR00000394
			32		Trp	CCA	tdbR00000484
			32		Ini	CAU	tdbR00000510-11
			32	Um	Gln	CUG	tdbR00000332
			32		Gln	U*UG	tdbR00000333
			32		Pro	CGG	tdbR00000623
	TrmL	yibK	34	Cm	Leu	CmAA	tdbR00000595
			34	cmnm^5^Um	Leu	cmnm^5^UmAA	tdbR00000220
*H. volcanii*	aTrmJ	HVO_2906	32	Cm	Lys	U*UU	tdbR00000175
					Lys	ac^4^CUU	tdbR00000176
					Trp	CmCA	tdbR00000478
					Tyr	GUA	tdbR00000541
	sRNA/aFib		34	Cm	Met	CmAU	tdbR00000271
			34	Cm	Trp	CmCA	tdbR00000478
			39	Um	Trp	CmCA	tdbR00000478
	aTrm56	HVO_1173	56	Cm	All known tRNAs		
*S. cerevisiae*	Trm13	YOL125w	4	Cm	Gly	GCC	tdbR00000129
			4	Cm	Pro	U*GG	tdbR00000323-4
			4	Am	His	GUG	tdbR00000145
	Trm3	YDL112w	18	Gm	Leu	CAA	tdbR00000249
			18		Leu	UAG	tdbR00000250
			18		Leu	UAA	tdbR00000251
			18		Trp	CmCA	tdbR00000494
			18		Ser	IGA	tdbR00000407-8
			18		Ser	U*GA	tdbR00000406
			18		His	GUG	tdbR00000145
			18		Tyr	GψA	tdbR00000555
	Trm7/Trm732	YBR061C/YMR259c	32	Cm	Trp	CmCA	tdbR00000494
			32		Phe	GmAA	tdbR00000083-4
			32		Leu	UAA	tdbR00000251
	Trm7/Trm734	YBR061C/Rtt10	34	Cm	Trp	CmCA	tdbR00000494
			34	Gm	Phe	GmAA	tdbR00000083-4
			34	cmnm^5^Um	Lys	cmnm^5^UmUU	tdbR00000193
	Trm44	YPL030w	44	Um	Ser	IGA	tdbR00000407-8
			44		Ser	U*GA	tdbR00000406
*H. sapiens*	hTrm13	CCDC76	4	Cm	Gly	CCC	tdbR00000135
			4	Um	Gly	GCC	tdbR00000136
	hTrm3	TARBP1	18	Gm	Asn	NUG	tdbR00000306
			18		Gln	U*UG	tdbR00000345
			18		Gln	U*UG	tdbR00000346
			18		Ser	UGA	tdbR00000428
	hTrm7	FTSJ1	32	Cm	Phe	GmAA	tdbR00000103
			32		Asn	NUG	tdbR00000306
			32		Gln	U*UG	tdbR00000345
			32		Gln	U*UG	tdbR00000346
			32	Um	Ala	IGC	tdbR00000016
			32		Ala	IGC	tdbR00000017
			34	Cm	Met	CmAU	tdbR00000289
			34	Gm	Phe	GmAA	tdbR00000103
	unknown		39	ψm	Gly	CCC	tdbR00000135
	hTrm44	METTL19	44	Um	Leu	NAA	tdbR00000269
			44		Ser	UGA	tdbR00000428
	unknown		54	m^5^Um	Glu	CUC	tdbR00000062

tRNA reference numbers are from tRNA database (tRNAdb). U*: unknown modified uridine; Q: queuosine; I: inosine; ψ: pseudouridine; N: unknown residue; other modified nucleotides abbreviated according to their conventional names. Trm: tRNA methyltransferase; aTrm: archaeal tRNA methyltransferase; hTrm: human tRNA methyltransferase; sRNA: small RNA; aFib: archaeal fibrillarin.

**Table 2:** Calculated ScoreMean and MethScore for known 2′-*O*-methylated positions in *E. coli* and *S. cerevisiae* tRNAs extracted from WT and the strain lacking tRNA:Gm18-MTase (TrmH or TRM3).

*Escherichia coli*									
				WT *E. coli*		ΔTrmH *E. coli*		Difference	
tdbR Number	tRNA	Modification	Position *	ScoreMean	MethScore	ScoreMean	MethScore	ScoreMean	MethScore
tdbR00000160	tRNA^Ile^(k^2^CAU)	Gm18	18	0.92	0.68	0.29	−0.37	**0.63**	**1.05**
tdbR00000332	tRNA^Gln^(CUG)	Gm18	18	0.97	0.85	0.94	0.79	0.03	0.06
tdbR00000333	tRNA^Gln^(U*UG)	Gm18	18	0.81	0.7	0.76	0.67	0.05	0.04
tdbR00000390	tRNA^Ser^(CGA)	Gm18	18	0.6	0.25	0.24	−0.3	**0.35**	**0.55**
tdbR00000392-393	tRNA^Ser^(GGA)	Gm18	18	0.86	0.76	0.26	0.32	**0.6**	**0.44**
tdbR00000394	tRNA^Ser^(cmo^5^UGA)	Gm18	18	0.98	0.88	0.94	0.8	0.04	0.08
tdbR00000545-546	tRNA^Tyr^(QUA)	Gm18	18	0.93	0.84	0.35	0.17	**0.58**	**0.67**
tdbR00000220	tRNA^Leu^(cmnm^5^UmAA)	Gm18	19	0.87	0.42	0.46	−0.78	**0.42**	**1.21**
tdbR00000221	tRNA^Leu^(CAG)	Gm18	19	0.97	0.89	0.35	−0.02	**0.62**	**0.9**
tdbR00000222	tRNA^Leu^(GAG)	Gm18	19	0.99	0.95	0.25	−0.48	**0.74**	**1.44**
tdbR00000276	tRNA^Met^(ac^4^CAU)	Gm18	19	0.58	0.42	0.75	0.44	−0.17	−0.02
tdbR00000595	tRNA^Leu^(CmAA)	Gm18	19	0.93	0.9	0.73	0.67	0.19	0.22
tdbR00000332	tRNA^Gln^(CUG)	Um32	32	0.97	0.85	0.94	0.79	0.03	0.06
tdbR00000333	tRNA^Gln^(U*UG)	Um32	32	0.81	0.7	0.76	0.67	0.05	0.04
tdbR00000484	tRNA^Trp^(CCA)	Cm32	33	0.99	0.92	0.99	0.91	0	0.01
tdbR00000394	tRNA^Ser^(cmo^5^UGA)	Cm32	34	0.98	0.88	0.94	0.8	0.04	0.08
tdbR00000510-511	tRNA^Met^_i_(CAU)	Cm32	34	0.97	0.84	0.97	0.85	0	−0.02
tdbR00000623	tRNA^Pro^(CGG)	Um32	34	0.98	0.93	0.97	0.92	0	0.01
tdbR00000595	tRNA^Leu^(CmAA)	Cm34	36	1	0.94	0.99	0.88	0.01	0.05
tdbR00000220	tRNA^Leu^(cmnm^5^UmAA)	cmnm^5^Um34	36	1	0.94	0.98	0.81	0.02	0.13
***Saccharomyces cerevisae***									
				**WT *S. cerevisiae***		**ΔTRM3 *S. cerevisiae***		**Difference**	
**tdbR Number**	**tRNA**	**Modification**	Position *	**ScoreMean**	**MethScore**	**ScoreMean**	**MethScore**	**ScoreMean**	**MethScore**
tdbR00000129	tRNA^Gly^(GCC)	Cm4	5	0.89	0.63	0.93	0.76	−0.04	−0.13
tdbR00000323-4	tRNA^Pro^(U*GG)	Cm4	5	0.95	0.83	0.95	0.81	0.01	0.02
tdbR00000145	tRNA^His^(GUG)	Am5	6	0.98	0.89	0.96	0.85	0.01	0.03
tdbR00000249	tRNA^Leu^(CAA)	Gm18	18	0.99	0.89	0.84	0.03	0.15	**0.87**
tdbR00000250	tRNA^Leu^(UAG)	Gm18	18	0.93	0.84	−0.35	−0.46	**1.28**	**1.29**
tdbR00000251	tRNA^Leu^(UAA)	Gm18	18	0.93	0.86	−0.16	−0.14	**1.10**	**1.01**
tdbR00000406	tRNA^Ser^(U*GA)	Gm18	18	0.91	0.85	0.75	0.57	**0.15**	**0.29**
tdbR00000407-8	tRNA^Ser^(IGA)	Gm18	18	0.95	0.81	−0.35	−0.80	**1.30**	**1.61**
tdbR00000494	tRNA^Trp^(CmCA)	Gm18	18	0.93	0.81	0.26	−0.05	**0.67**	**0.87**
tdbR00000145	tRNA^His^(GUG)	Gm18	19	0.91	0.80	−0.98	−0.84	**1.89**	**1.64**
tdbR00000555	tRNA^Tyr^(G_ψ_A)	Gm18	19	0.96	0.92	0.09	0.13	**0.87**	**0.79**
tdbR00000494	tRNA^Trp^(CmCA)	Cm32	32	0.99	0.86	0.99	0.88	0.00	−0.02
tdbR00000083-4	tRNA^Phe^(GmAA)	Cm32	33	0.98	0.79	0.98	0.81	0.00	−0.03
tdbR00000251	tRNA^Leu^(UAA)	Cm32	34	0.92	0.55	0.95	0.69	−0.03	−0.14
tdbR00000494	tRNA^Trp^(CmCA)	Cm34	34	0.99	0.87	0.99	0.89	0.00	−0.03
tdbR00000083-4	tRNA^Phe^(GmAA)	Gm34	35	0.96	0.74	0.93	0.63	0.03	0.10
tdbR00000193	tRNA^Lys^(cmnm^5^UmUU)	cmnm^5^Um34	35	0.64	0.30	0.58	0.26	0.06	0.04
tdbR00000406	tRNA^Ser^(U*GA)	Um44	45	0.93	0.86	0.93	0.83	−0.01	0.03
tdbR00000407-8	tRNA^Ser^(IGA)	Um44	45	0.90	0.78	0.86	0.71	0.04	0.07

tdbR identifiers are taken from tRNAdb [66]; when the collapsed sequence is used, both numbers are indicated. * The position corresponds to the real position of the signal in the sequence of tRNA; thus it appears at position +1 to the 2′-*O*-methylated residue. U*: unknown modified uridine; Q: queuosine; I: inosine; ψ: pseudouridine. Important changes in ScoreMean and MethScore indicating disappearence of Gm18 are bolded.

## Supplementary Materials

The following are available online at http://www.mdpi.com/2218-273X/7/1/11/S1, Supplementary Materials and Methods, Figure S1: Levenshtein distances between tRNA gene (tDNA) sequences from *E. coli* and *S. cerevisiae*, Figure S2: False-positive hits observed in the *E. coli* transfer RNA (tRNA) cleavage profiles, Figure S3: Quantification of Gm in *E. coli* tRNA^Met^ by liquid chromatography coupled to tandem mass-spectrometry (LC-MS/MS), Figure S4: False-positive hits observed in the *S. cerevisiae* tRNA cleavage profiles, Table S1: Initial and collapsed *E. coli* tDNA dataset, Table S2: Initial and collapsed *S. cerevisiae* tDNA dataset, Table S3 :Number of sequencing reads used for the RiboMethSeq analysis of different samples.
